# Epigallocatechin gallate alleviates high-fat diet-induced hepatic lipotoxicity by targeting mitochondrial ROS-mediated ferroptosis

**DOI:** 10.3389/fphar.2023.1148814

**Published:** 2023-03-21

**Authors:** Shi-Bin Ding, Xiao-Lei Chu, Yu-Xuan Jin, Jin-Jin Jiang, Xiao Zhao, Min Yu

**Affiliations:** Jiangsu Vocational College of Medicine, Yancheng, China

**Keywords:** epigallocatechin gallate, mitochondrial ROS, ferroptosis, high-fat diet, hepatic lipotoxicity

## Abstract

**Background:** Non-alcoholic fatty liver disease (NAFLD) is a chronic advanced liver disease that is highly related to metabolic disorders and induced by a high-fat diet (HFD). Recently, epigallocatechin gallate (EGCG) has been regarded as a protective bioactive polyphenol in green tea that has the ability to protect against non-alcoholic fatty liver disease, but the molecular mechanism remains poorly deciphered. Ferroptosis plays a vital role in the progression of non-alcoholic fatty liver disease, but experimental evidence of ferroptosis inhibition by epigallocatechin gallate is limited. Hence, our study aimed to investigate the effect and mechanisms of epigallocatechin gallate on hepatic ferroptosis to mitigate hepatic injury in high-fat diet-fed mice.

**Methods:** Fifty male C57BL/6 mice were fed either a standard chow diet (SCD), a high-fat diet, or a high-fat diet and administered epigallocatechin gallate or ferrostatin-1 (a ferroptosis-specific inhibitor) for 12 weeks. Liver injury, lipid accumulation, hepatic steatosis, oxidative stress, iron overload, and ferroptosis marker proteins were examined. *In vitro*, steatotic L-02 cells were used to explore the underlying mechanism.

**Results:** In our research, we found that epigallocatechin gallate notably alleviated liver injury and lipid accumulation, oxidative stress, hepatic steatosis, decreased iron overload and inhibited ferroptosis in a high-fat diet-induced murine model of non-alcoholic fatty liver disease. In vitro experiments, using ferrostatin-1 and a mitochondrial reactive oxygen species (MtROS) scavenger (Mito-TEMPO), we found that epigallocatechin gallate remarkably alleviated oxidative stress and inhibited ferroptosis by reducing the level of mitochondrial reactive oxygen species in steatotic L-02 cells.

**Conclusion:** Taken together, our results revealed that epigallocatechin gallate may exert protective effects on hepatic lipotoxicity by inhibiting mitochondrial reactive oxygen species-mediated hepatic ferroptosis. Findings from our study provide new insight into prevention and treatment strategies for non-alcoholic fatty liver disease pathological processes.

## 1 Introduction

Non-alcoholic fatty liver disease (NAFLD) is the most common chronic liver disease worldwide. Recently, NAFLD has been renamed metabolic dysfunction-associated fatty liver disease (MAFLD), which includes simple steatosis to steatohepatitis (NASH) and can potentially develop into cirrhosis and hepatocarcinoma (Barchetta et al., 2020). Currently, it has been estimated that the morbidity of NAFLD in the world’s general population is approximately 17%–33% (Spahis et al., 2017; Tang et al., 2021). In Western countries, NAFLD has a morbidity rate of up to 30%, with obesity causing an increase in the number of cases (Younossi et al., 2016; Nagasaki et al., 2020). In China, 29.2% of the population suffers from NAFLD, and the burden is projected to grow significantly in the coming decades (Zhou et al., 2019). Thus, this epidemic of NAFLD has become a global public health problem, and it is critical to develop a therapeutic strategy for targeting NAFLD.

The current theory on NAFLD pathogenesis is the “two-hit hypothesis,” which is widely accepted to explain the pathogenesis mechanism of NAFLD (Day and James, 1998; Rada et al., 2020). In that hypothesis, excess triglycerides accumulated in hepatocytes (hepatic steatosis) is present in the initial stage of NAFLD, which acts as the “first fit,” and the subsequent lipid peroxidation, oxidative stress and inflammation serve as the “second fit.” A high-fat diet (HFD) and diet-induced obesity lead to hepatic fat accumulation, which is deemed a major cause of NAFLD. Currently, no clinical drugs have been approved for the treatment of NAFLD, and the management of NAFLD mainly relies on a healthy diet and lifestyle changes. It has been confirmed that fat accumulation in the liver can cause oxidative stress, increase lipid peroxidation and trigger inflammatory responses, which promote hepatic injury (Gomez-Zorita et al., 2020; Park et al., 2020). Therefore, natural antioxidants (such as natural polyphenols) are one of the most effective strategies for the treatment of NAFLD (Salomone et al., 2016). Green tea is one of the most consumed beverages in Asian countries and contains many phytochemicals, such as tea polyphenols. Epigallocatechin gallate (EGCG) is a kind of tea polyphenol that exhibits various salutary effects, such as antioxidant, anti-inflammatory, hypocholesterolemic, and antihypertensive effects (Chen et al., 2008; Kim et al., 2014). Mounting evidence suggests that EGCG exerts beneficial effects on NAFLD patients, and the protective mechanism may include regulating lipid metabolism and suppressing hepatic stellate cell activation (Kuzu et al., 2008), attenuating fibrosis, oxidative stress, and inflammation (Xiao et al., 2014), and improving the gut microbiota (Ning et al., 2020). Recently, several studies have reported that EGCG could inhibit ferroptosis in human intestinal epithelial HIEC cells (Xie et al., 2020) and in murine pancreatic cells (Kose et al., 2019), but the effect and molecular mechanism of EGCG on ferroptosis in NAFLD remain unclear. Ferroptosis is a novel form of non-apoptotic cell death that depends on iron (Dixon et al., 2012). It has been demonstrated that the Fenton reaction catalyzed by intracellular labile iron causes lipid peroxidation in cells, which is considered the main cause of ferroptosis (Dixon et al., 2012; Yang et al., 2016). As a newly discovered form of cell death, ferroptosis plays an important role in the development and progression of NAFLD (Wang et al., 2022). *In vivo* and *in vitro* experiments have confirmed that hepatic ferroptosis suppression protects against NASH (Qi et al., 2020; Jiang et al., 2022). In light of this, we hypothesized that EGCG exerts a beneficial effect on diet-induced NAFLD by suppressing hepatic ferroptosis to alleviate hepatic lipotoxicity.

In the present study, we found that the preventive effect of EGCG on hepatic lipotoxicity was involved in ferroptosis in HFD-induced NAFLD. Furthermore, we confirmed that EGCG may protect against hepatic injury by reducing MtROS-mediated ferroptosis in HFD-fed mice and steatotic hepatocytes. These findings provide novel insight into EGCG for the prevention and treatment of patients with NAFLD.

## 2 Materials and methods

### 2.1 Chemicals and reagents

EGCG (purity ≥98%) was purchased from Solarbio (Beijing, China). Fetal bovine serum (FBS) and DMEM were obtained from Gibco (United States). A total protein extraction kit was purchased from KeyGEN BioTech. (Jiangsu, China). A BCA protein assay kit was purchased from Dingguo Changsheng Biotechnology (Beijing, China). Rabbit anti-glutathione peroxidase 4 (GPX4), rabbit anti-ACSL4, rabbit anti-GAPDH, and goat anti-IgG (H + L)-HRP secondary antibodies were obtained from Proteintech Bio, Inc. (Wuhan, China). Ferrostatin-1 (Fer-1) was purchased from MedChemExpress LLC. Normal diet and high-fat diet (HFD) were obtained from TROPHIC Animal Feed High-tech Co., Ltd. (China).

### 2.2 Animal care and experimental treatments

Fifty male C57BL/6 mice (6 weeks old, 17–22 g) were purchased from Vital River Laboratory Animal Technology Co., Ltd. (Beijing, China). Animals were housed at 23°C ± 2°C with 12-h light/dark cycles and specific pathogen-free conditions, with free access to food and water. Animal experiments were approved and conducted in accordance with the guidelines of the Animal Care and Use Committee of Jiangsu Vacational College of Medicine.

After adaptive feeding, mice were randomly assigned to five groups (*n* = 10 per group). The details of the groups are as follows: 1) the normal diet (ND) group in which mice were fed ND (18% calories from fat); 2) the HFD group in which mice were fed HFD (60% calories from fat); 3) the HFD-EGCG/L group in which mice received 20 mg/kgbw EGCG by oral gavage daily during HFD feeding; 4) the HFD-EGCG/H group in which mice received 100 mg/kgbw EGCG by oral gavage daily during HFD feeding; and 5) the HFD-Fer-1 group in which mice received intraperitoneal injection of Fer-1 at 1 mg/kg. bw every 3 days during HFD feeding. Mice in the EGCG treatment groups were supplemented with EGCG (20 and 100 mg/kgbw) for 12 weeks. Meanwhile, mice in the ND group and the HFD group were orally gavaged with deionized water daily.

### 2.3 Tissue preparation

After treatment, mice were deeply anesthetized with intravenous pentobarbital injection (20 mg/kg). Before sacrifice, blood was obtained by cardiac puncture, and serum was separated by centrifugation at 3,000 rpm for 20 min at 4°C. Liver tissues were rapidly removed, weighed and then fixed in 4% formaldehyde for histopathologic assessment or snap-frozen in liquid nitrogen and then stored at −80°C for further experiments.

### 2.4 Cell culture conditions and establishment of the steatotic hepatocyte model

Normal human hepatocytes (L-02 cells) were grown in Dulbecco’s modified Eagle’s medium (DMEM) supplemented with 10% fetal bovine serum, 100 U/mL penicillin and 100 μg/mL streptomycin in an atmosphere containing 5% CO_2_ at 37°C. To establish the steatotic hepatocyte model, a 0.5 mmol/L free fatty acid (FFA) mixture (oleic acid: palmitic acid ratio, 2:1) was added to L-02 cells for 24 h, and then the cells were used for experiments when they reached 80% confluence.

### 2.5 Cell viability assay

After treatment with EGCG for 24 h, L-02 cells were incubated with the Cell Counting Kit-8 (CCK-8) (Beyotime Biotechnology, Shanghai, China) in wells according to the instruments, and the absorbance was measured at 450 nm.

### 2.6 Biochemical analysis

Serum alanine aminotransferase (ALT) and aspartate aminotransferase (AST) levels were determined using commercial enzymatic kits (Nanjing Jiancheng Bioengineering, Inc., Nanjing, China). Total lipids in the liver were extracted according to the protocol of a previous study (Ding et al., 2018), and TG in the liver was determined using a commercial assay kit (Sangon Biotech, Inc., Shanghai, China).

### 2.7 Iron measurement

The iron concentration in liver tissues and cell lysates was determined with a commercial Iron Assay kit (MLBIO, Inc., Shanghai, China) according to the manufacturer’s instructions.

### 2.8 Oxidative stress measurement

4-Hydroxynonenal (4-HNE) in liver tissues and cell lysates was measured with a commercial ELISA kit (MLBIO, Inc., Shanghai, China), and experiments were performed according to the manufacturer’s instructions. The content of reduced glutathione (GSH)/GSSG (oxidized glutathione) in liver tissues of mice and cell lysates were determined by commercial assay kits (Nanjing Jiancheng Bioengineering, Inc., Nanjing, China).

### 2.9 Histopathological assessments of livers

Fresh liver samples from the right lobe of livers were fixed in 4% paraformaldehyde at room temperature for 24 h, embedded in paraffin and sectioned into 5 μm-thick sections. Liver slices were stained with hematoxylin-eosin (H&E) and Oil Red O for histological examinations. Hepatic steatosis of mice was calculated using a point-counting method as previously described (Catta-Preta et al., 2011).

### 2.10 Flow cytometric analysis of lipid reactive oxygen species

The L-02 cells were pretreated with EGCG (25 and 50 μmol/L) for 8 h and then incubated with the FFA mixture for 24 h to establish the steatotic hepatocyte model. L-02 cells were incubated with 5 μmol/L BODIPY 581/591C11 molecular probes (Invitrogen, United States) at 37°C for 30 min in the dark as previously described (Jiang et al., 2022). Then, the lipid reactive oxygen species were measured using flow cytometric analysis.

### 2.11 Lipid droplet immunofluorescence staining in hepatocytes

L-02 cells were cultured with FFA in six-well plates for 48 h, and then the cells were processed with two concentrations of EGCG and stained with Lipi-Red (DOJINDO, Beijing, China) for 30 min using a standard protocol. To visualize the cellular distribution of lipid droplets, confocal microscopy (Zeiss, Germany) was used to capture immunofluorescence images of lipid droplets in FFA-treated L-02 cells.

### 2.12 Flow cytometric analysis of MtROS in cells

After treatment with EGCG or Mito-TEMPO, L-02 cells were incubated in the dye MitoSox Red Mitochondrial Superoxide indicator (5 μM, Thermo, United States) in phosphate buffered saline (PBS) for 30 min at 37°C in the dark. The cells were washed with PBS three times, and mitochondrial ROS levels were determined using flow cytometry.

### 2.13 Western blot analysis

Total proteins were extracted from liver tissues and L-02 cells using RIPA lysis buffer (Beyotime Biotechnology, Shanghai, China). The protein concentration of the samples was determined using a BCA protein assay kit (DINGGUO, Bio Co., Ltd., Beijing, China) according to the manufacturer’s protocols. Protein samples were electrophoresed on a 10% SDS-polyacrylamide gel and transferred to a nitrocellulose (NC) membrane. Membranes were blocked with 5% nonfat milk in Tris buffered saline buffer (TBS) and incubated with primary antibodies against GPX4, COX-2, ACSL4, and GAPDH overnight at 4°C. Membranes were washed with TBS with Tween-20 and then incubated with the corresponding secondary antibody for 2 h at room temperature. Protein signals were detected using an ECL detection system (Tanon, Shanghai, China).

### 2.14 Statistical analysis

Data are expressed as the mean ± standard deviation (SD). All data were analyzed with SPSS 25.0 (SPSS, Chicago, IL, United States) by suitable statistical protocols. Comparisons between groups were analyzed using one-way analysis of variance (one-way ANOVA) followed by *post hoc* analysis (Bonferroni posttest), and a *p*-value less than 0.05 (*p* < 0.05) was considered statistically significant.

## 3 Results

### 3.1 EGCG alleviated hepatic steatosis and injury in HFD-fed mice

**FIGURE 1:**
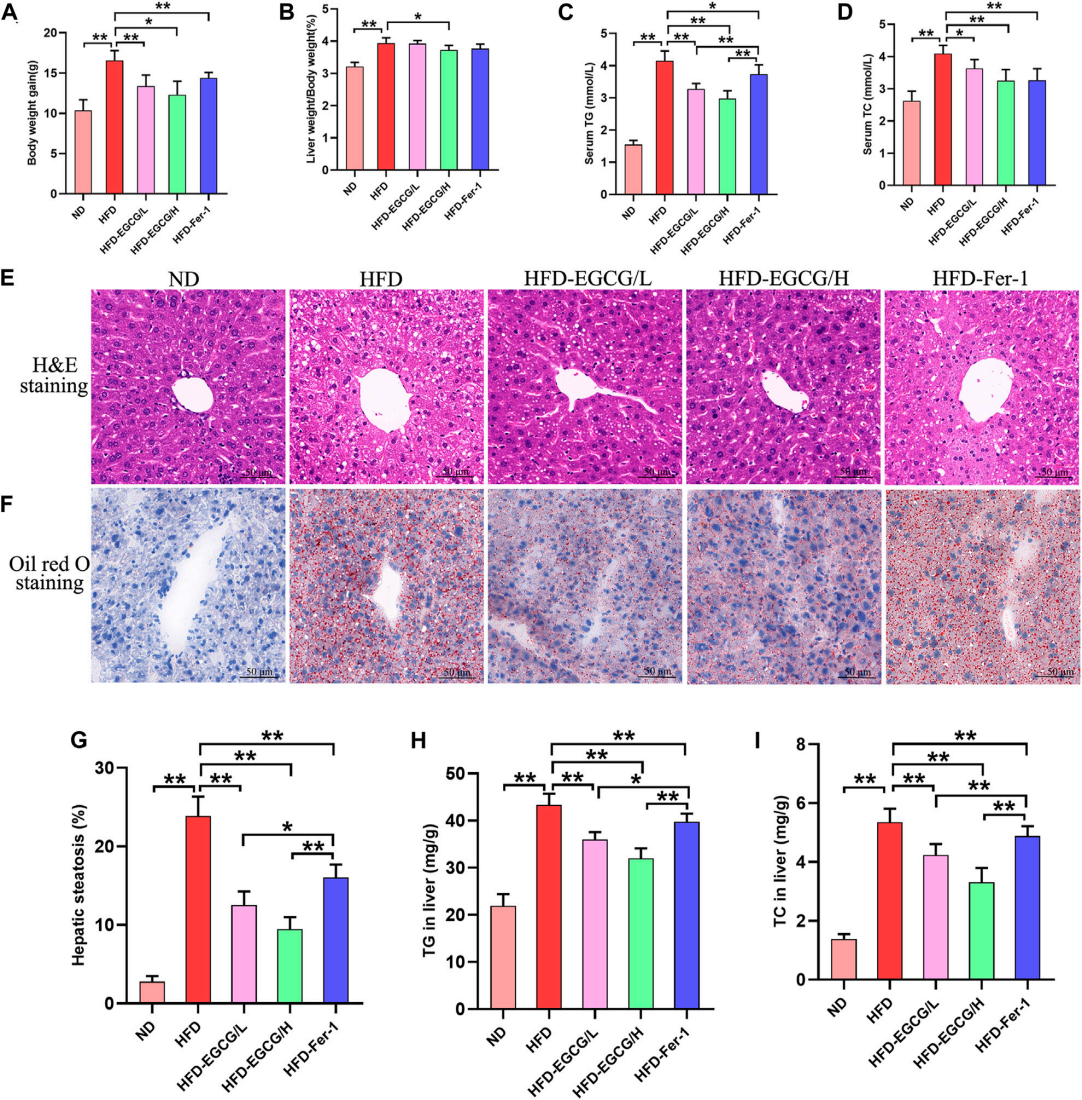
EGCG alleviated lipid metabolism parameters and hepatic injury in HFD-fed mice. **(A)** Body weight gain (*n* = 10). **(B)** Liver weight/body weight (*n* = 10). **(C)** Serum TG (*n* = 10). **(D)** Serum TC (*n* = 10). **(E)** H&E staining of the liver (scale bars = 50 μm). **(F)** Oil Red O staining of liver (scale bars = 50 μm). **(G)** The quantitation of hepatic steatosis (*n* = 5). **(H)** TG in the liver (*n* = 10). **(I)** TC in liver (*n* = 10). Data are expressed as the mean ± SD. **, *p* < 0.01 and *, *p* < 0.05.

As presented in Figures 1A–D, the body weight gain, liver weight/body weight, serum TG and serum TC were significantly increased in HFD-fed mice compared to the mice in the ND group, and EGCG treatment significantly decreased body weight gain, liver weight/body weight, serum TG, and serum TC in HFD-fed mice. To assess hepatic steatosis and histopathological changes after HFD feeding, H&E staining and Oil Red O staining of liver sections were performed (Figures 1E, F). We found that the liver sections of mice from the HFD group showed extensive vacuolar degeneration of hepatocytes and lipid droplet accumulation, suggesting HFD-induced hepatic steatosis in mice. Moreover, both EGCG treatment and Fer-1 treatment significantly alleviated lipid droplet accumulation in hepatocytes in HFD-fed mice. Visualization and quantification analysis of hepatic steatosis and TG found that two doses of EGCG intervention and Fer-1 treatment significantly reduced hepatic steatosis percentage, TG and TC in liver tissues in HFD-fed mice (Figures 1G–I). In addition, the hepatic steatosis percentage and the levels of serum TG, TG, and TC in liver tissues in the EGCG-treated groups were significantly lower than those in the HFD-Fer-1 group (Figures 1C, G, I). These results indicated that EGCG intervention improved hepatic steatosis and liver injury in HFD-fed mice.

### 3.2 EGCG reduced the level of mitochondrial ROS and improved liver injury in liver tissues of HFD-fed mice

**FIGURE 2:**
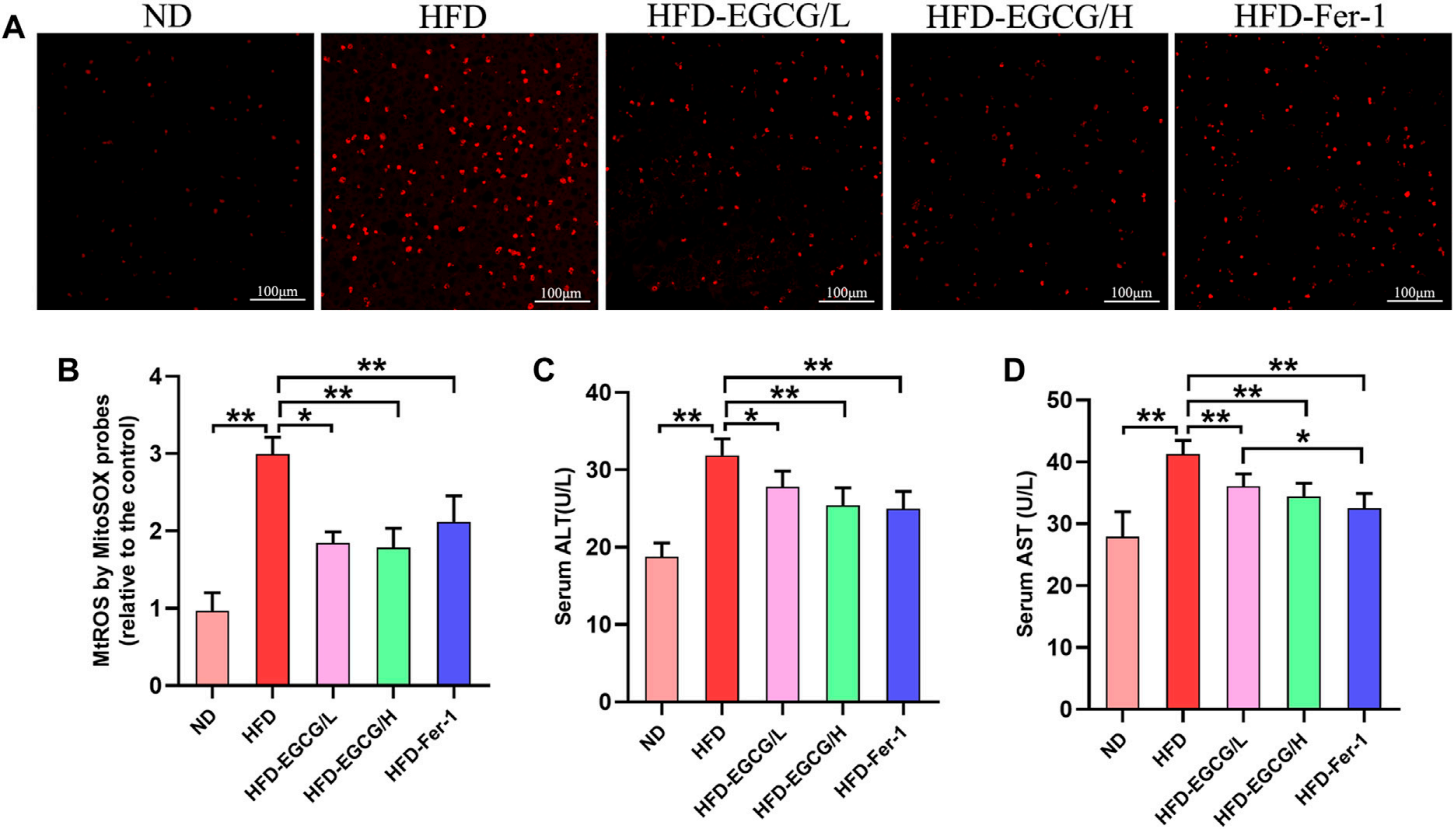
EGCG reduced the level of mitochondrial ROS and liver injury in liver tissues of HFD-fed mice. **(A)** Representative images show MitoSOX red fluorescence in liver tissues (scale bar = 100 μm). **(B)** Results of relative MitoSOX fluorescence density (*n* = 5). **(C)** Serum ALT (*n* = 10). **(D)** Serum AST (*n* = 10). Data are expressed as the mean ± SD. **, *p* < 0.01 and *, *p* < 0.05.

To confirm whether EGCG could reduce HFD-induced mitochondrial ROS in the liver, we observed and quantified the levels of mitochondrial ROS in liver tissues by MitoSOX red fluorescence (Figures 2A, B). Compared with the control group, the relative MitoSOX fluorescence density was significantly increased in the HFD group. Moreover, the relative MitoSOX fluorescence density in the EGCG-treated groups and the Fer-1-treated group was obviously lower than that in the HFD group (Figures 2A, B). Furthermore, EGCG and Fer-1 treatment significantly decreased the levels of serum ALT and serum AST in HFD-fed mice (Figures 2C, D). These results indicated that EGCG supplementation could protect against HFD-induced mitochondrial ROS in the liver and alleviate liver injury.

### 3.3 EGCG reduced iron content, oxidative stress and ferroptosis in the livers of HFD-fed mice

**FIGURE 3:**
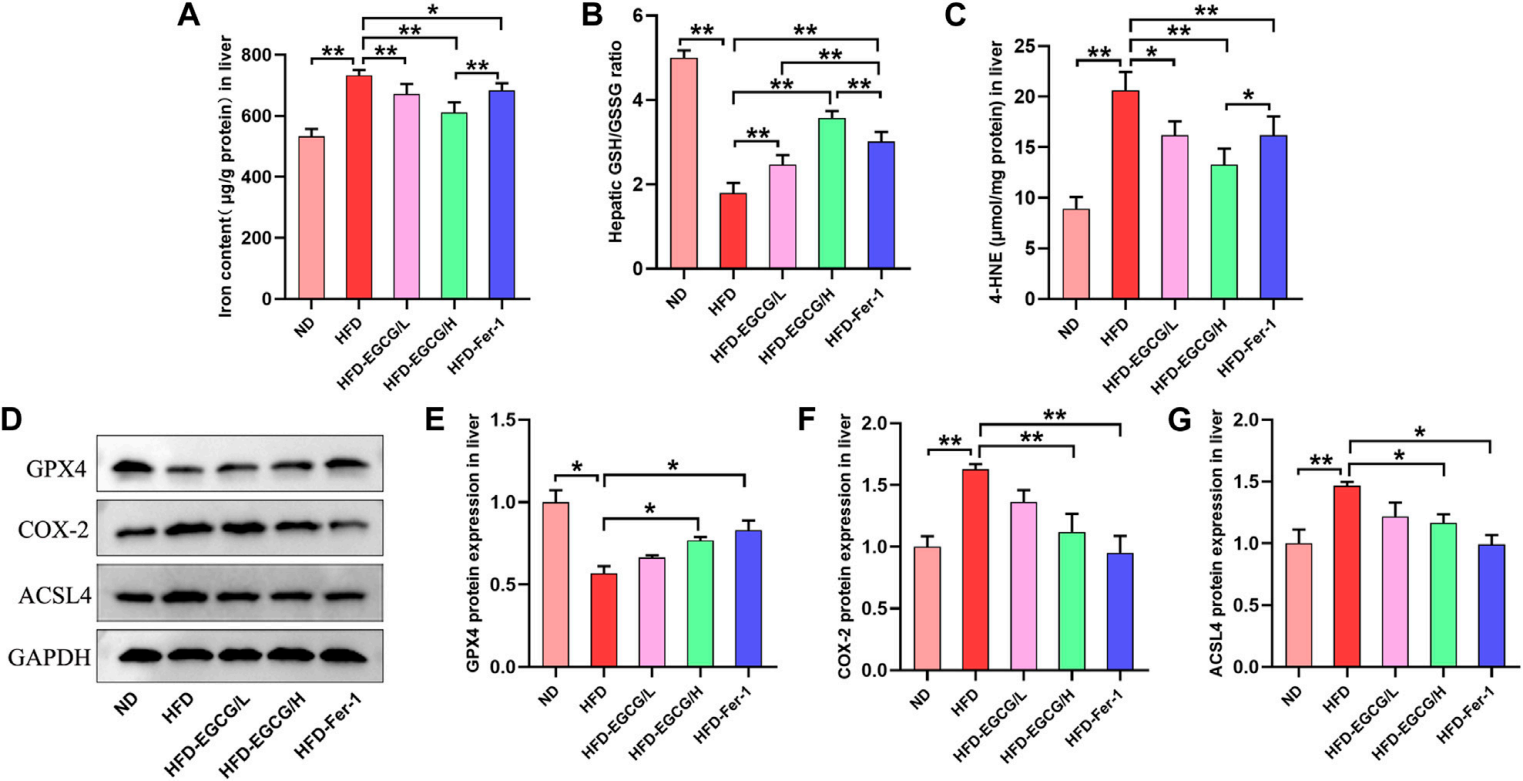
EGCG reduced iron content, oxidative stress and ferroptosis in the livers of HFD-fed mice. **(A)** Liver iron content (*n* = 6). **(B, C)** The levels of GSH/GSSG and 4-HNE in the liver (*n* = 6). **(D)** Representative Western blot images. **(E–G)** The expression of ferroptosis-related proteins (GPX4, COX-2 and ACSL4) in the liver (*n* = 3). Data are expressed as the mean ± SD. **, *p* < 0.01 and *, *p* < 0.05.

Next, we assessed the effects of EGCG on hepatic iron content, oxidative stress and ferroptosis *in vivo*. Compared to the control group, the hepatic iron content and the content of 4-HNE were obviously increased, and the ratio of GSH/GSSG was decreased in the HFD group mice (Figures 3A–C). These results suggest HFD-induced iron accumulation and oxidative stress in the liver. Both groups with doses of EGCG administration and the HFD-Fer-1 group showed markedly lower iron accumulation and oxidative stress in the liver (Figures 3A–C). Western blotting results showed that the protein expression levels of COX-2 and ACSL4 in the livers of the HFD group mice were significantly higher than those in the livers of the ND group mice, and the protein expression of GPX4 in the livers of the HFD group mice was significantly lower than that in the livers of the ND group mice (Figures 3D–G). In addition, EGCG supplementation (100 mg/kgbw) and Fer-1 treatment apparently increased the protein expression of GPX4 and markedly decreased the protein expression of COX-2 and ACSL4 in the livers of HFD-fed mice (Figures 3D–G). These results indicated that HFD feeding caused hepatic ferroptosis in mice, which was alleviated by EGCG supplementation and Fer-1 treatment.

### 3.4 EGCG reduced lipid droplet accumulation in steatotic L-02 cells

**FIGURE 4:**
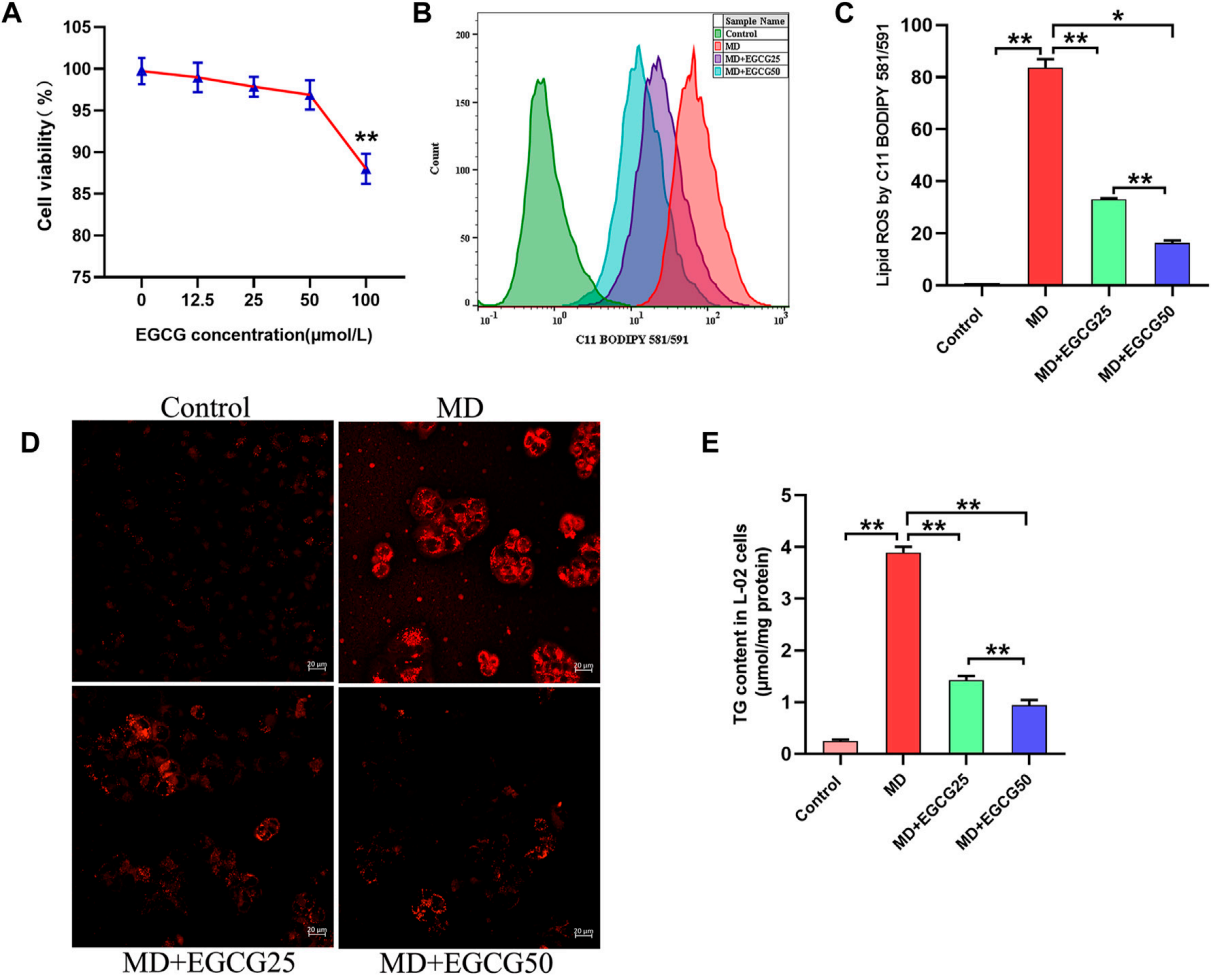
EGCG reduced lipid accumulation in steatotic L-02 cells. **(A)** The effect of EGCG on the viability of L-02 cells. **(B)** Representative fluorescence intensity images of cellular lipid ROS. **(C)** Flow cytometric analysis of fluorescence intensity. **(D)** Representative images of red fluorescence (lipid droplets) by Lipi-Red in L-02 cells (scale bars = 50 μm). **(E)** TG content. Data are expressed as the mean ± SD from three dependent experiments. **, *p* < 0.01 and *, *p* < 0.05.

To evaluate the effect of EGCG on the cell viability of L-02 cells, cells were cultured and treated with different concentrations of EGCG (0, 12.5, 25, 50, and 100 μmol/L) for 24 h, and then the cell viability was determined by CCK-8 assays. As shown in Figure 4A, the viability of L-02 cells showed a significant decline at EGCG concentrations of 100 μmol/L. Thus, 25 μmol/L, and 50 μmol/L EGCG were used in subsequent *in vitro* experiments. As shown in Figures 4B, C, EGCG treatment significantly decreased the level of cellular lipid ROS in steatotic L-02 cells. As shown in Figure 4D, FFA mixtures induced lipid droplet accumulation in L-02 cells, indicating that the steatotic hepatocyte model was established. Moreover, treatment with EGCG decreased lipid droplet accumulation in steatotic L-02 cells compared to the steatotic hepatocyte model (Figure 4D). Moreover, the TG content of L-02 cells in the MD group was significantly higher than that in the control group, and the TG content in the MD group was significantly lower than that in the MD-EGCG/25 group and the MD-EGCG/50 group (Figure 4E). These results together suggest that EGCG can reduce lipid droplet accumulation in the FFA mixture-induced steatotic hepatocyte model.

### 3.5 EGCG reduced oxidative stress and ferroptosis in steatotic L-02 cells

**FIGURE 5:**
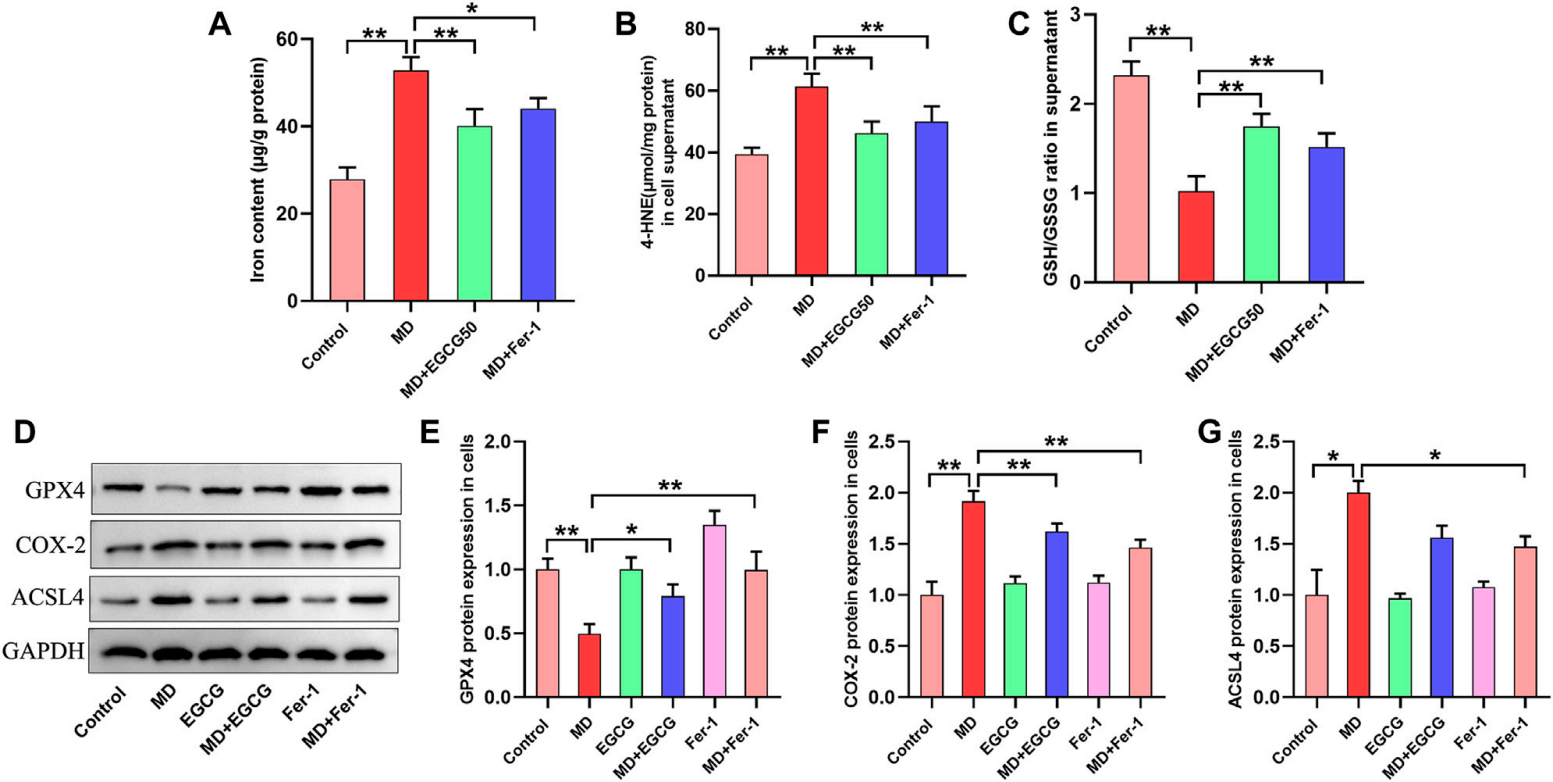
EGCG reduced oxidative stress, iron overload and ferroptosis in steatotic L-02 cells. **(A, B)** The levels of GSH/GSSG and 4-HNE. **(C)** Iron content. **(D)** Representative Western blot images. **(E–G)** The protein expression of ferroptosis-related proteins (GPX4, COX-2, and ACSL4) in L-02 cells. Data are expressed as the mean ± SD from three dependent experiments. **, *p* < 0.01 and *, *p* < 0.05.

Subsequently, we investigated the effect of EGCG and Fer-1 on oxidative stress and ferroptosis in steatotic L-02 cells. As shown in Figures 5A–C, a decreased GSH/GSSG ratio and increased iron and 4-HNE contents were observed in the steatotic hepatocytes. Moreover, EGCG treatment and Fer-1 treatment obviously increased the GSH/GSSG ratio and significantly decreased the iron and 4-HNE contents in steatotic hepatocytes (Figures 5A–C). Moreover, downregulated protein expression of GPX4 and upregulated protein expression of COX-4 and ACSL4 were observed in steatotic hepatocytes (Figures 5D–G). Additionally, Que treatment and Fer-1 treatment obviously increased GPX4 protein expression and decreased COX-4 and ACSL4 protein expression in steatotic hepatocytes (Figures 5D–G). These results indicate that EGCG can suppress ferroptosis in steatotic hepatocytes *in vitro*.

### 3.6 EGCG inhibited ferroptosis by reducing MtROS in steatotic L-02 cells

**FIGURE 6:**
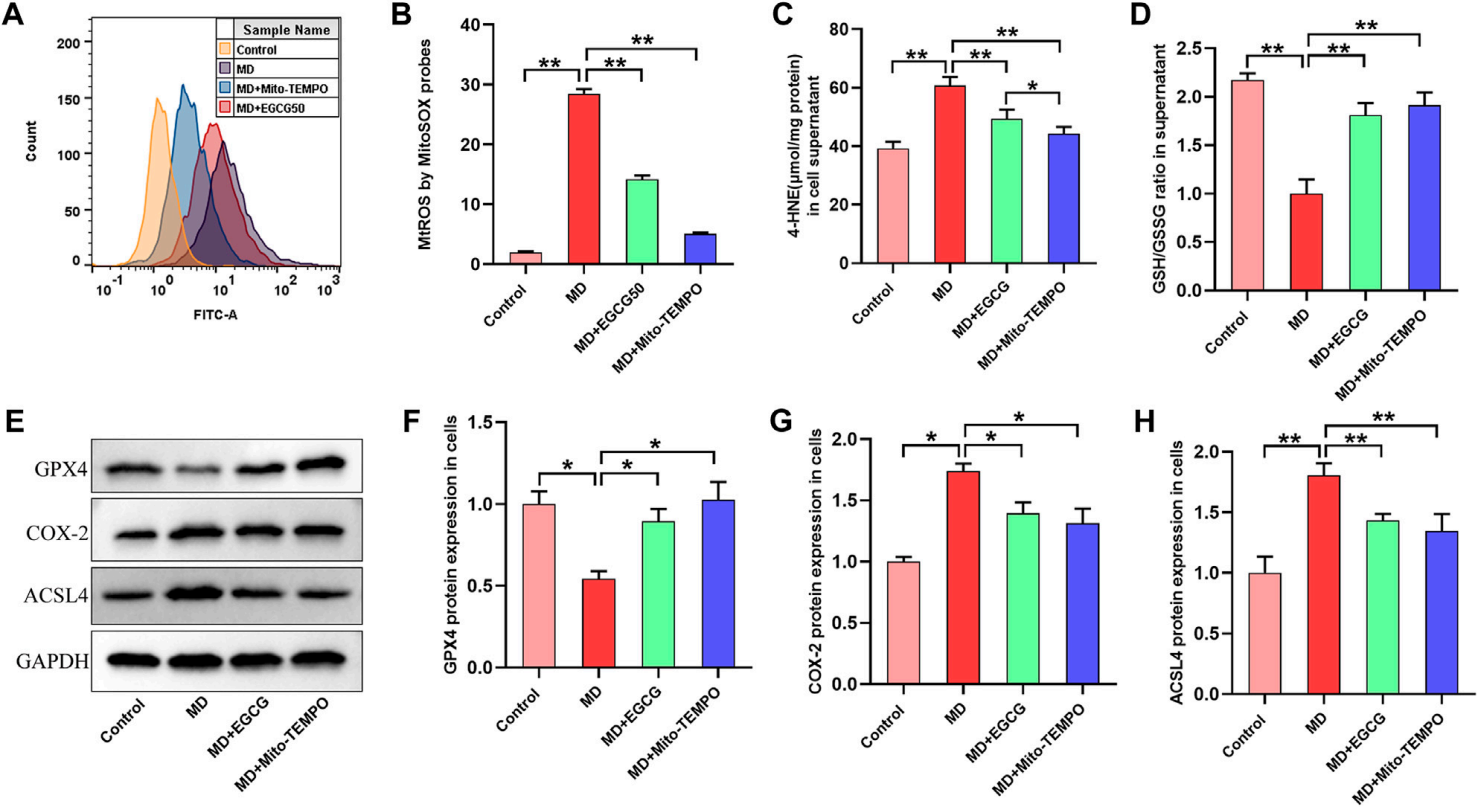
EGCG inhibited ferroptosis by reducing MtROS in steatotic L-02 cells. **(A)** Fluorescence intensity images of MtROS by MitoSOX probes determined by flow cytometry. **(B)** Flow cytometry results of MtROS. **(C, D)** The levels of GSH/GSSG and 4-HNE. **(E)** Representative images of Western blot. **(F–H)** The protein expression of ferroptosis-related proteins (GPX4, COX-2, and ACSL4). Data are expressed as the mean ± SD from three dependent experiments. **, *p* < 0.01 and *, *p* < 0.05.

To investigate the mechanism by which EGCG suppresses ferroptosis in steatotic hepatocytes, we determined the level of MtROS, oxidative stress and ferroptosis-related proteins in pretreated steatotic L-02 cells with EGCG (50 μmol/L) and Mito-TEMPO (the MtROS scavenger). As depicted in Figures 6A, B, the level of MtROS in the steatotic L-02 cells was obviously increased compared to that in the control group. Interestingly, the overproduction of MtROS in steatotic L-02 cells was significantly reduced after pretreatment with EGCG and Mito-TEMPO (Figures 6A, B). We found significantly decreased 4-HNE and an elevated GSH/GSSG ratio in steatotic L-02 cells pretreated with EGCG and Mito-TEMPO (Figures 6C, D), suggesting reduced oxidative stress in steatotic hepatocytes under EGCG and Mito-TEMPO treatment. As shown in Figures 6E–H, we observed downregulated GPX4 protein expression and upregulated COX-2 and ACSL4 protein expression in steatotic L-02 cells compared to the control group. As expected, EGCG pretreatment significantly increased GPX4 expression and decreased COX-2 and ACSL4 expression in steatotic L-02 cells (Figures 6E–H). Consistently, Mito-TEMPO treatment also obviously rescued these changes in ferroptosis-related protein expression in steatotic L-02 cells (Figures 6E–H). Together, these results indicate that EGCG treatment can suppress cellular ferroptosis in FFA-mixture-induced steatotic L-02 cells by reducing MtROS.

## 4 Discussion

Excessive fatty acid (FFA) influx to hepatocytes causes lipotoxicity, which is involved in NAFLD progression. In the present study, we found that HFD consumption increased MtROS generation and oxidative stress and triggered ferroptosis, ultimately leading to hepatic lipid accumulation and lipotoxicity. Mechanistically, we demonstrated that HFD-induced ferroptosis depended upon the increased MtROS in hepatocytes. Importantly, EGCG treatment apparently alleviated HFD-induced hepatic lipotoxicity by targeting mitochondrial ROS-mediated ferroptosis.

HFD is an environmental risk factor for human health that can induce lipid accumulation in the liver, accelerate hepatic lipotoxicity and ultimately cause NAFLD. Accumulating evidence from clinical trials and systematic reviews has confirmed the beneficial functions of green tea and its components in preventing and managing NAFLD (Sakata et al., 2013; Mahmoodi et al., 2020). The most abundant and bioactive component of green tea is EGCG, which has been thoroughly studied. An increasing amount of evidence has revealed that EGCG exerts therapeutic effects on NAFLD that are mainly associated with improvement of lipid metabolism and reduction of oxidative stress and inflammation, as well as suppression of hepatic fibrosis (Xiao et al., 2014; Chen et al., 2018). The term “lipotoxicity” refers to an accumulation of lipid that exceeds the capacity of the cells to store it, resulting in hepatocyte dysfunction (Mendez-Sanchez et al., 2018) and abnormal functioning of the tissues (such as liver, muscle, heart, pancreas, arteries, etc.) (Engin, 2017). A previous *in vitro* study reported that EGCG treatment improved insulin resistance in HepG2 cells possibly through alleviating lipotoxicity and inflammation (Zhang et al., 2018). The effect and molecular mechanism of EGCG on hepatic lipotoxicity are still unclear. Abnormal accumulation of lipids in the liver causes hepatic steatosis. In this study, we found that HFD feeding increased the ratio of liver weight to body weight and caused liver injury and lipid accumulation, indicating HFD-induced hepatic steatosis. It has been reported that EGCG improved serum lipids and reduced inflammation associated with aging in HFD-fed obese rats (Yuan et al., 2020). Du et al. (2021) reported that EGCG (25 mg/kg/day and 50 mg/kg/day) administration for 8 weeks significantly attenuated body and liver weight and reduced hepatic TG accumulation and the hepatic steatosis score in a murine model of NAFLD. Consistent with the results of previous studies, our study found that treatment with various concentrations of EGCG (20 mg/kg/day and 100 mg/kg/day) for 12 weeks improved liver injury and hepatic steatosis in HFD-fed mice, suggesting two doses of EGCG improved hepatic lipotoxicity in a HFD-induced murine model of NAFLD.

Regulated cell death of hepatocytes determines the severity and outcome of hepatic injury. Ferroptosis is a newly discovered iron-dependent programmed cell death that is different from other types of cell death, such as apoptosis, necrosis and autophagy (Zhang et al., 2020). A previous study reported that ferroptosis inhibition attenuated iron overload-induced liver injury and fibrosis (Wu et al., 2021). Ning et al. (2020) revealed that ferroptosis is involved in the progression of NASH in a methionine/choline-deficient diet-induced NASH mouse model. Moreover, an increasing number of studies have revealed that suppression of hepatic ferroptosis improves HFD-induced NAFLD (Yang et al., 2020; Zhu et al., 2021). These findings indicated that to prevent the progression of NASH and its associated lipotoxicity, ferroptosis might serve as a repair regulator against liver injury in the early stage of NASH. In addition, oxidative stress plays an important role in NASH, which can cause ferroptosis in hepatocytes. Therefore, we investigated the effect of EGCG treatment on oxidative stress and ferroptosis in the liver of a NAFLD murine model. Our study showed that HFD caused iron accumulation and increased oxidative stress and ferroptosis in the liver, and EGCG treatment significantly reversed these changes in HFD-fed mice. Taken together, our data indicated that EGCG treatment improved hepatic lipotoxicity in the NAFLD murine model by inhibiting ferroptosis.

The regulatory molecular mechanisms of hepatic ferroptosis are complicated. To reveal the mechanism of ferroptosis involved in the progression of NAFLD, a steatotic hepatocyte model was established by FFA mixtures, as shown by an abnormal accumulation of lipids. In the present study, our results showed that a significant dose-dependent reduction in TG in steatotic L-02 cells was observed after two doses of EGCG treatment, which is in accordance with the results of immunofluorescence staining of lipid droplets in hepatocytes. *In vitro*, we further verified that EGCG is functionally similar to Fer-1 (a ferroptosis inhibitor) in the regulation of hepatic ferroptosis, which effectively inhibited ferroptosis in steatotic L-02 cells.

Oxidative stress caused by excessive ROS production increases lipid peroxidation and inflammatory cytokines, which further promotes hepatic lipid accumulation and inflammation. The mitochondrion is an important cellular organelle that plays a crucial role in energy metabolism and is a major site for ROS production (Dan Dunn et al., 2015). NAFLD has been shown to increase the ROS levels (Videla et al., 2004). Recent studies have confirmed that mtROS in hepatocytes promote the development of NAFLD (Simoes et al., 2018). Nevertheless, little is known about the signaling pathways linked to MtROS and the progression stages of NAFLD. Considering that mitochondria play a vital role in cell death regulation (Chipuk et al., 2021), and some important metabolic processes in mitochondria (such as the tricarboxylic acid cycle) are involved in ferroptosis (Gao et al., 2019), mitochondria might participate in regulating ferroptosis. Therefore, our study further investigated whether EGCG could inhibit ferroptosis in steatotic hepatocytes. *In vitro*, our research found that the production of MtROS was increased in steatotic hepatocytes. Meanwhile, we observed that EGCG reduced the generation of MtROS in steatotic hepatocytes, indicating that EGCG has the ability to scavenge hepatic MtROS. Notably, administration of mito-TEMPO and EGCG obviously suppressed ferroptosis in FFA-induced steatotic L-02 hepatocytes, confirmed that EGCG is functionally similar to mito-TEMPO in the regulation of hepatocytes ferroptosis. Collectively, our study first demonstrated that EGCG, as an antioxidant, alleviated HFD-induced hepatic lipotoxicity by scavenging ROS to suppress ferroptosis in hepatocytes.

In conclusion, we demonstrated that EGCG can protect against HFD-induced hepatic lipotoxicity in a murine model of NAFLD. *In vitro*, we confirmed that EGCG exerts a protective effect on lipid accumulation, oxidative stress and ferroptosis by reducing MtROS in steatotic hepatocytes. Thus, our study provides an effective EGCG therapeutic strategy for the intervention of hepatic steatosis and injury.

## Data Availability

The original contributions presented in the study are included in the article/supplementary material, further inquiries can be directed to the corresponding author.

## References

[B1] Barchetta I. Cimini F. A. Cavallo M. G. (2020). Vitamin D and metabolic dysfunction-associated fatty liver disease (MAFLD): An update. Nutrients 12 (11), 3302. 10.3390/nu12113302 33126575 PMC7693133

[B2] Catta-Preta M. Mendonca L. S. Fraulob-Aquino J. Aguila M. B. Mandarim-de-Lacerda C. A. (2011). A critical analysis of three quantitative methods of assessment of hepatic steatosis in liver biopsies. Virchows Arch. 459 (5), 477–485. 10.1007/s00428-011-1147-1 21901430

[B3] Chen C. Liu Q. Liu L. Hu Y. Y. Feng Q. (2018). Potential biological effects of (-)-Epigallocatechin-3-gallate on the treatment of nonalcoholic fatty liver disease. Mol. Nutr. Food Res. 62 (1), 1700483. 10.1002/mnfr.201700483 28799714 PMC6120134

[B4] Chen D. Milacic V. Chen M. S. Wan S. B. Lam W. H. Huo C. (2008). Tea polyphenols, their biological effects and potential molecular targets. Histol. Histopathol. 23 (4), 487–496. 10.14670/HH-23.487 18228206 PMC3763709

[B5] Chipuk J. E. Mohammed J. N. Gelles J. D. Chen Y. (2021). Mechanistic connections between mitochondrial biology and regulated cell death. Dev. Cell 56 (9), 1221–1233. 10.1016/j.devcel.2021.03.033 33887204 PMC8102388

[B6] Dan Dunn J. Alvarez L. A. Zhang X. Soldati T. (2015). Reactive oxygen species and mitochondria: A nexus of cellular homeostasis. Redox Biol. 6, 472–485. 10.1016/j.redox.2015.09.005 26432659 PMC4596921

[B7] Day C. P. James O. F. (1998). Steatohepatitis: A tale of two "hits. Gastroenterology 114 (4), 842–845. 10.1016/s0016-5085(98)70599-2 9547102

[B8] Ding S. Yu L. An B. Zhang G. Yu P. Wang Z. (2018). Combination effects of airborne particulate matter exposure and high-fat diet on hepatic fibrosis through regulating the ROS-endoplasmic reticulum stress-TGFβ/SMADs axis in mice. Chemosphere 199, 538–545. 10.1016/j.chemosphere.2018.02.082 29455124

[B9] Dixon S. J. Lemberg K. M. Lamprecht M. R. Skouta R. Zaitsev E. M. Gleason C. E. (2012). Ferroptosis: An iron-dependent form of nonapoptotic cell death. Cell 149 (5), 1060–1072. 10.1016/j.cell.2012.03.042 22632970 PMC3367386

[B10] Du Y. Paglicawan L. Soomro S. Abunofal O. Baig S. Vanarsa K. (2021). Epigallocatechin-3-Gallate dampens non-alcoholic fatty liver by modulating liver function, lipid profile and macrophage polarization. Nutrients 13 (2), 599. 10.3390/nu13020599 33670347 PMC7918805

[B11] Engin A. B. (2017). What is lipotoxicity? Adv. Exp. Med. Biol. 960, 197–220. 10.1007/978-3-319-48382-5_8 28585200

[B12] Gao M. Yi J. Zhu J. Minikes A. M. Monian P. Thompson C. B. (2019). Role of mitochondria in ferroptosis. Mol. Cell 73 (2), 354–363.e3. 10.1016/j.molcel.2018.10.042 30581146 PMC6338496

[B13] Gomez-Zorita S. Gonzalez-Arceo M. Trepiana J. Aguirre L. Crujeiras A. B. Irles E. (2020). Comparative effects of pterostilbene and its parent compound resveratrol on oxidative stress and inflammation in steatohepatitis induced by high-fat high-fructose feeding. Antioxidants (Basel) 9 (11), 1042. 10.3390/antiox9111042 33114299 PMC7690896

[B14] Jiang J. J. Zhang G. F. Zheng J. Y. Sun J. H. Ding S. B. (2022). Targeting mitochondrial ROS-mediated ferroptosis by quercetin alleviates high-fat diet-induced hepatic lipotoxicity. Front. Pharmacol. 13, 876550. 10.3389/fphar.2022.876550 35496312 PMC9039018

[B15] Kim H. S. Quon M. J. Kim J. A. (2014). New insights into the mechanisms of polyphenols beyond antioxidant properties; lessons from the green tea polyphenol, epigallocatechin 3-gallate. Redox Biol. 2, 187–195. 10.1016/j.redox.2013.12.022 24494192 PMC3909779

[B16] Kose T. Vera-Aviles M. Sharp P. A. Latunde-Dada G. O. (2019). Curcumin and (-)- epigallocatechin-3-gallate protect murine MIN6 pancreatic beta-cells against iron toxicity and erastin-induced ferroptosis. Pharm. (Basel) 12 (1), 26. 10.3390/ph12010026 PMC6469157 30736288

[B17] Kuzu N. Bahcecioglu I. H. Dagli A. F. Ozercan I. H. Ustundag B. Sahin K. (2008). Epigallocatechin gallate attenuates experimental non-alcoholic steatohepatitis induced by high fat diet. J. Gastroenterol. Hepatol. 23 (8), e465–e470. 10.1111/j.1440-1746.2007.05052.x 17683497

[B18] Mahmoodi M. Hosseini R. Kazemi A. Ofori-Asenso R. Mazidi M. Mazloomi S. M. (2020). Effects of green tea or green tea catechin on liver enzymes in healthy individuals and people with nonalcoholic fatty liver disease: A systematic review and meta-analysis of randomized clinical trials. Phytother. Res. 34 (7), 1587–1598. 10.1002/ptr.6637 32067271

[B19] Mendez-Sanchez N. Cruz-Ramon V. C. Ramirez-Perez O. L. Hwang J. P. Barranco-Fragoso B. Cordova-Gallardo J. (2018). New aspects of lipotoxicity in nonalcoholic steatohepatitis. Int. J. Mol. Sci. 19 (7), 2034. 10.3390/ijms19072034 30011790 PMC6073816

[B20] Nagasaki A. Sakamoto S. Chea C. Ishida E. Furusho H. Fujii M. (2020). Odontogenic infection by Porphyromonas gingivalis exacerbates fibrosis in NASH via hepatic stellate cell activation. Sci. Rep. 10 (1), 4134. 10.1038/s41598-020-60904-8 32139740 PMC7058079

[B21] Ning K. Lu K. Chen Q. Guo Z. Du X. Riaz F. (2020). Epigallocatechin gallate protects mice against methionine-choline-deficient-diet-induced nonalcoholic steatohepatitis by improving gut microbiota to attenuate hepatic injury and regulate metabolism. ACS Omega 5 (33), 20800–20809. 10.1021/acsomega.0c01689 32875214 PMC7450495

[B22] Park M. Yoo J. H. Lee Y. S. Park E. J. Lee H. J. (2020). Ameliorative effects of black ginseng on nonalcoholic fatty liver disease in free fatty acid-induced HepG2 cells and high-fat/high-fructose diet-fed mice. J. Ginseng Res. 44 (2), 350–361. 10.1016/j.jgr.2019.09.004 32148418 PMC7031749

[B23] Qi J. Kim J. W. Zhou Z. Lim C. W. Kim B. (2020). Ferroptosis affects the progression of nonalcoholic steatohepatitis via the modulation of lipid peroxidation-mediated cell death in mice. Am. J. Pathol. 190 (1), 68–81. 10.1016/j.ajpath.2019.09.011 31610178

[B24] Rada P. Gonzalez-Rodriguez A. Garcia-Monzon C. Valverde A. M. (2020). Understanding lipotoxicity in NAFLD pathogenesis: Is CD36 a key driver? Cell Death Dis. 11 (9), 802. 10.1038/s41419-020-03003-w 32978374 PMC7519685

[B25] Sakata R. Nakamura T. Torimura T. Ueno T. Sata M. (2013). Green tea with high-density catechins improves liver function and fat infiltration in non-alcoholic fatty liver disease (NAFLD) patients: A double-blind placebo-controlled study. Int. J. Mol. Med. 32 (5), 989–994. 10.3892/ijmm.2013.1503 24065295

[B26] Salomone F. Godos J. Zelber-Sagi S. (2016). Natural antioxidants for non-alcoholic fatty liver disease: Molecular targets and clinical perspectives. Liver Int. 36 (1), 5–20. 10.1111/liv.12975 26436447

[B27] Simoes I. C. M. Fontes A. Pinton P. Zischka H. Wieckowski M. R. (2018). Mitochondria in non-alcoholic fatty liver disease. Int. J. Biochem. Cell Biol. 95, 93–99. 10.1016/j.biocel.2017.12.019 29288054

[B28] Spahis S. Delvin E. Borys J. M. Levy E. (2017). Oxidative stress as a critical factor in nonalcoholic fatty liver disease pathogenesis. Antioxid. Redox Signal 26 (10), 519–541. 10.1089/ars.2016.6776 27452109

[B29] Tang G. Xu Y. Zhang C. Wang N. Li H. Feng Y. (2021). Green tea and epigallocatechin gallate (EGCG) for the management of nonalcoholic fatty liver diseases (NAFLD): Insights into the role of oxidative stress and antioxidant mechanism. Antioxidants (Basel) 10 (7), 1076. 10.3390/antiox10071076 34356308 PMC8301033

[B30] Wang S. Liu Z. Geng J. Li L. Feng X. (2022). An overview of ferroptosis in non-alcoholic fatty liver disease. Biomed. Pharmacother. 153, 113374. 10.1016/j.biopha.2022.113374 35834990

[B31] Wu A. Feng B. Yu J. Yan L. Che L. Zhuo Y. (2021). Fibroblast growth factor 21 attenuates iron overload-induced liver injury and fibrosis by inhibiting ferroptosis. Redox Biol. 46, 102131. 10.1016/j.redox.2021.102131 34530349 PMC8445902

[B32] Xiao J. Ho C. T. Liong E. C. Nanji A. A. Leung T. M. Lau T. Y. (2014). Epigallocatechin gallate attenuates fibrosis, oxidative stress, and inflammation in non-alcoholic fatty liver disease rat model through TGF/SMAD, PI3 K/Akt/FoxO1, and NF-kappa B pathways. Eur. J. Nutr. 53 (1), 187–199. 10.1007/s00394-013-0516-8 23515587

[B33] Xie L. W. Cai S. Zhao T. S. Li M. Tian Y. (2020). Green tea derivative (-)-epigallocatechin-3-gallate (EGCG) confers protection against ionizing radiation-induced intestinal epithelial cell death both *in vitro* and *in vivo* . Free Radic. Biol. Med. 161, 175–186. 10.1016/j.freeradbiomed.2020.10.012 33069855

[B34] Yang W. S. Kim K. J. Gaschler M. M. Patel M. Shchepinov M. S. Stockwell B. R. (2016). Peroxidation of polyunsaturated fatty acids by lipoxygenases drives ferroptosis. Proc. Natl. Acad. Sci. U. S. A. 113 (34), E4966–E4975. 10.1073/pnas.1603244113 27506793 PMC5003261

[B35] Yang Y. Chen J. Gao Q. Shan X. Wang J. Lv Z. (2020). Study on the attenuated effect of Ginkgolide B on ferroptosis in high fat diet induced nonalcoholic fatty liver disease. Toxicology 445, 152599. 10.1016/j.tox.2020.152599 32976958

[B36] Younossi Z. M. Blissett D. Blissett R. Henry L. Stepanova M. Younossi Y. (2016). The economic and clinical burden of nonalcoholic fatty liver disease in the United States and Europe. Hepatology 64 (5), 1577–1586. 10.1002/hep.28785 27543837

[B37] Yuan H. Li Y. Ling F. Guan Y. Zhang D. Zhu Q. (2020). The phytochemical epigallocatechin gallate prolongs the lifespan by improving lipid metabolism, reducing inflammation and oxidative stress in high-fat diet-fed obese rats. Aging Cell 19 (9), e13199. 10.1111/acel.13199 32729662 PMC7511879

[B38] Zhang Q. Yuan H. Zhang C. Guan Y. Wu Y. Ling F. (2018). Epigallocatechin gallate improves insulin resistance in HepG2 cells through alleviating inflammation and lipotoxicity. Diabetes Res. Clin. Pract. 142, 363–373. 10.1016/j.diabres.2018.06.017 29940201

[B39] Zhang X. Huang Z. Xie Z. Chen Y. Zheng Z. Wei X. (2020). Homocysteine induces oxidative stress and ferroptosis of nucleus pulposus via enhancing methylation of GPX4. Free Radic. Biol. Med. 160, 552–565. 10.1016/j.freeradbiomed.2020.08.029 32896601

[B40] Zhou F. Zhou J. Wang W. Zhang X. J. Ji Y. X. Zhang P. (2019). Unexpected rapid increase in the burden of NAFLD in China from 2008 to 2018: A systematic review and meta-analysis. Hepatology 70 (4), 1119–1133. 10.1002/hep.30702 31070259

[B41] Zhu Z. Zhang Y. Huang X. Can L. Zhao X. Wang Y. (2021). Thymosin beta 4 alleviates non-alcoholic fatty liver by inhibiting ferroptosis via up-regulation of GPX4. Eur. J. Pharmacol. 908, 174351. 10.1016/j.ejphar.2021.174351 34280397

